# Supplementary material for: Uniform Lithium Deposition Induced by ZnFx(OH)y for High-Performance Sulfurized Polyacrylonitrile-Based Lithium-Sulfur Batteries
Source: Polymers (Basel). 2022 Oct 24;14(21):4494. doi: 10.3390/polym14214494 (PMC9657706; doi:10.3390/polym14214494)
Supplement: Supplementary file 1 [file polymers-14-04494-s001.zip › polymers-1942104-supplementary.pdf]

Supporting Information

Uniform lithium deposition induced by $ZnF_x(OH)_y$ for high performance sulfurized polyacrylonitrile-based lithium-sulfur batteries

Wanming Teng[1,2], Yanyan Li[3], Ting Ma[1,2], Xiuyun Ren[4], Ding Nan[1,2]*, Jun Liu[2], Xiaohu Wang[2,5], Qin Yang[6], Jiaojiao Deng[6]*

[1] College of Chemistry and Chemical Engineering, Inner Mongolia University, Hohhot, 010021 China

[2] Inner Mongolia Key Laboratory of Graphite and Graphene for Energy Storage and Coating, School of Materials Science and Engineering, Inner Mongolia University of Technology, Hohhot, 010051 China

[3] State Key Laboratory of High-Performance Ceramics and Superfine Microstructures, Shanghai Institute of Ceramics, Chinese Academy of Sciences, 1295 Dingxi Road, Shanghai 200050, People's Republic of China

[4] College of Materials Science and Engineering, Qingdao University, Qingdao 266071, China

[5] Rising Graphite Applied Technology Research Institute, Chinese Graphite Industrial Park-Xinghe, Ulanqab, Inner Mongolia, 013650, China

[6] Shenzhen Key Laboratory on Power Battery Safety and Shenzhen Geim Graphene Center, Tsinghua Shenzhen International Graduate School (SIGS), Shenzhen, 518071 China

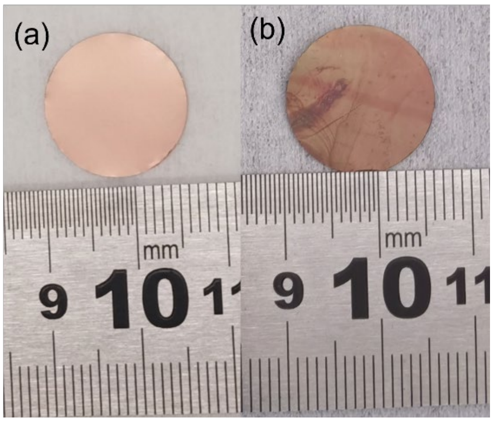

Figure S1 The Optical photographs of (a) bare Cu foil and (b) $ZnF_x(OH)_y$@Cu.

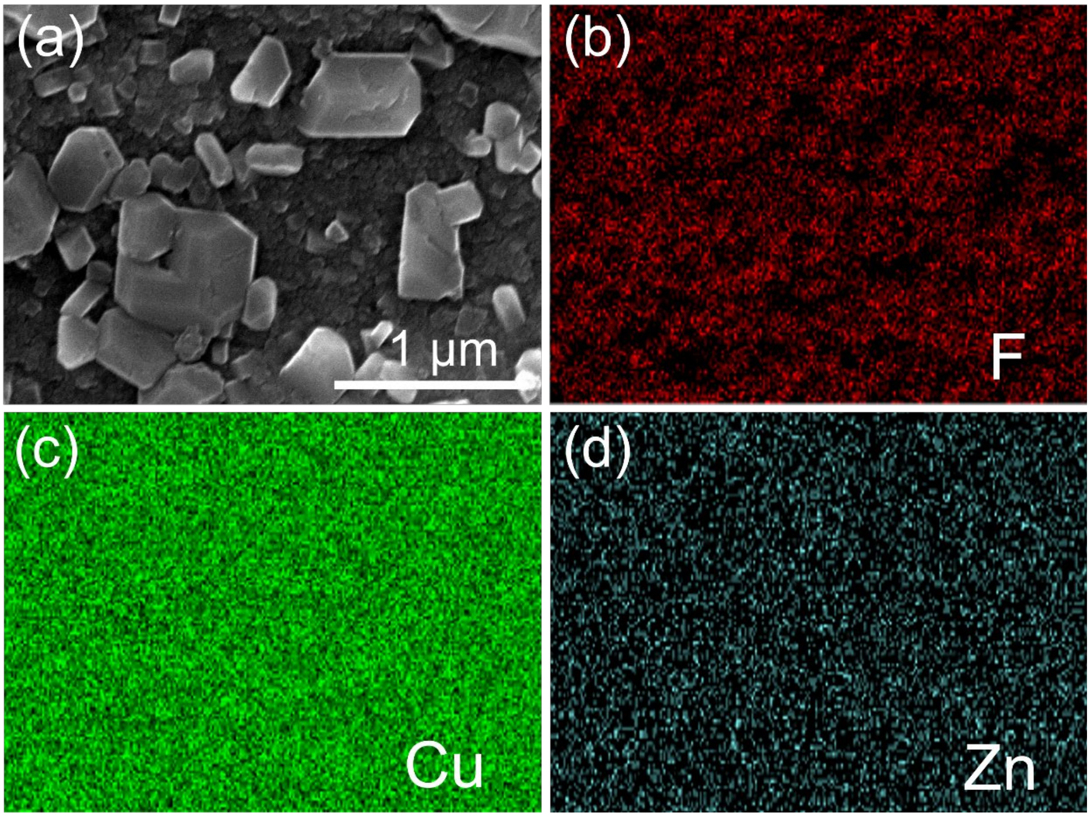

Figure S2 (a) SEM image of $ZnF_x(OH)_y$@Cu. (b-d) EDS elemental mapping images of $ZnF_x(OH)_y$@Cu (fluorine, zinc, copper).

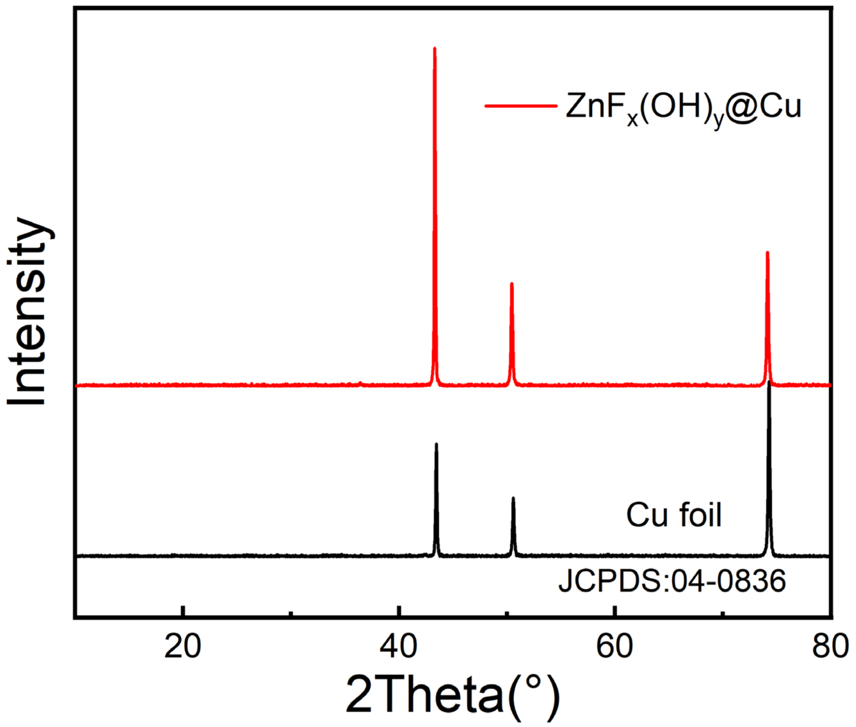

Figure S3 Comparison of XRD patterns of copper-based current collectors before and after the reaction.

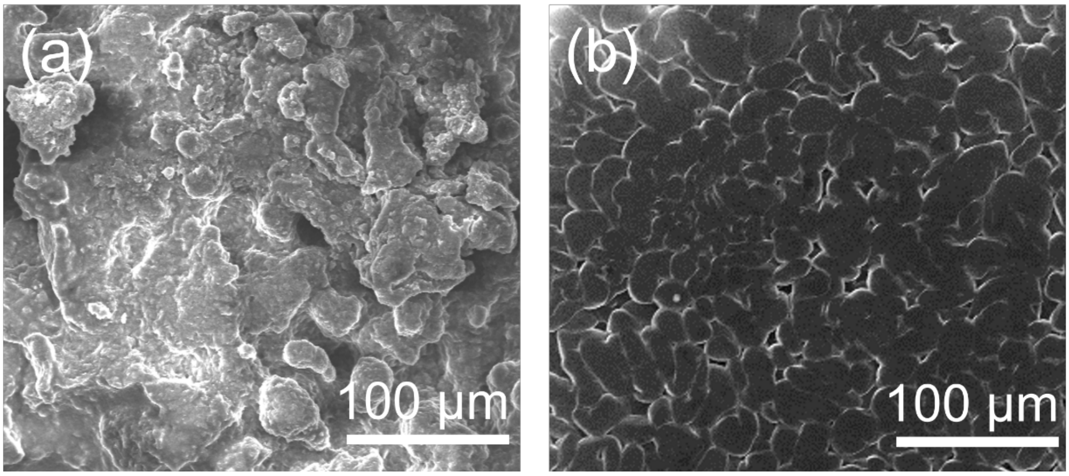

Figure S4 SEM image after lithium deposition. (a) Bare Cu (b) $ZnF_x(OH)_y$@Cu (current density: 0.5 mA cm^{-2}; deposition capacity: 1.0 mAh cm^{-2})

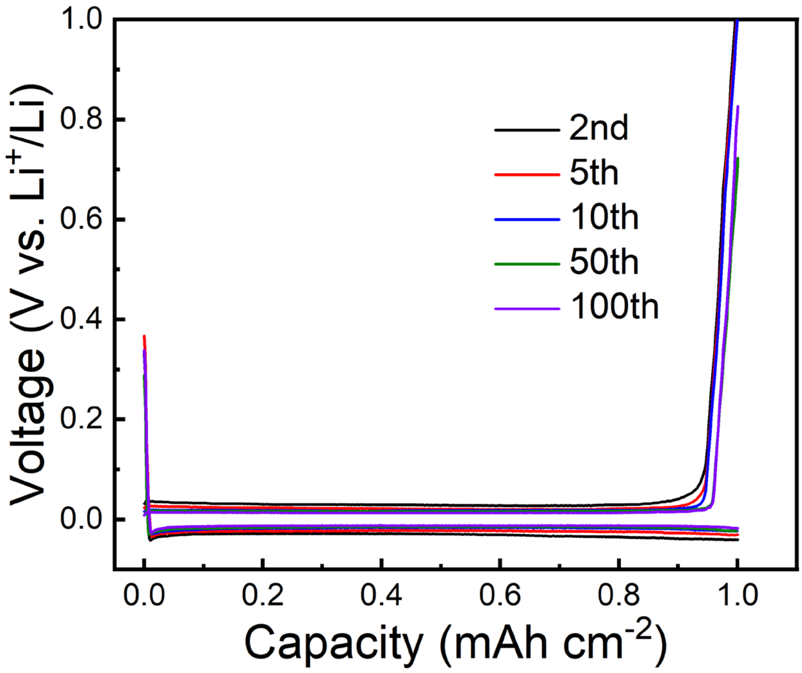

Figure S5 Electrochemical Li plating/stripping curves of the $ZnF_x(OH)_y$@Cu at 0.5 mA cm^{-2} with a specific capacity of 1 mAh cm^{-2}.

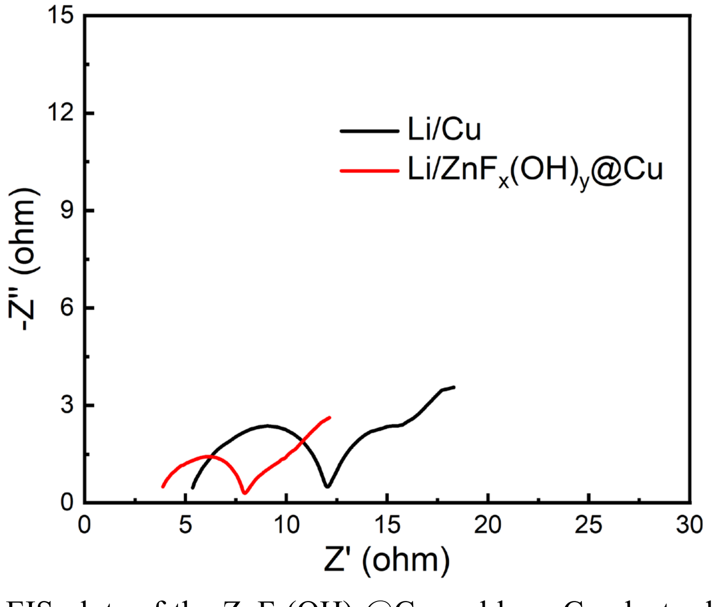

Figure S6 The EIS plots of the $ZnF_x(OH)_y$@Cu and bare Cu electrodes after the 5th cycles.

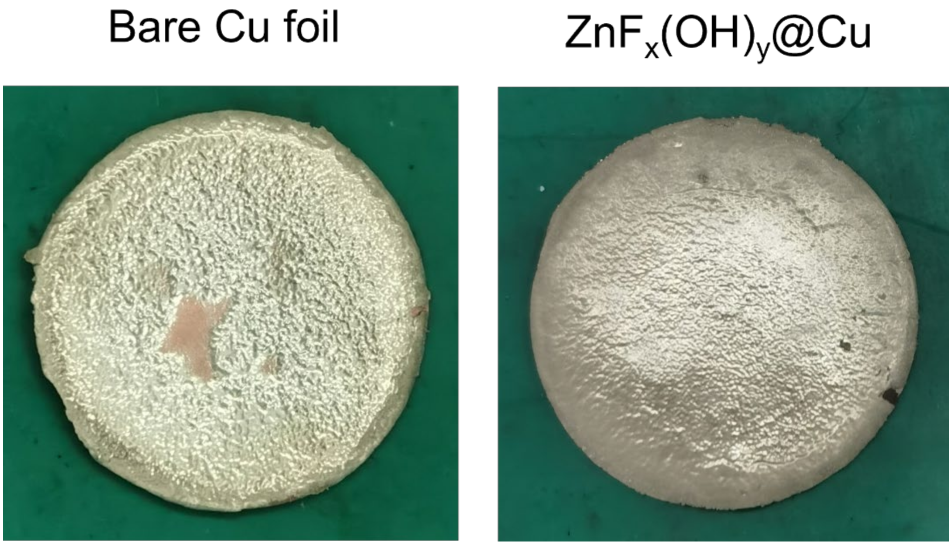

Figure S7. Photo images of the bare Cu foil and the $ZnF_x(OH)_y$@Cu foil after Li deposition at 6.0 mAh cm^{-2}.

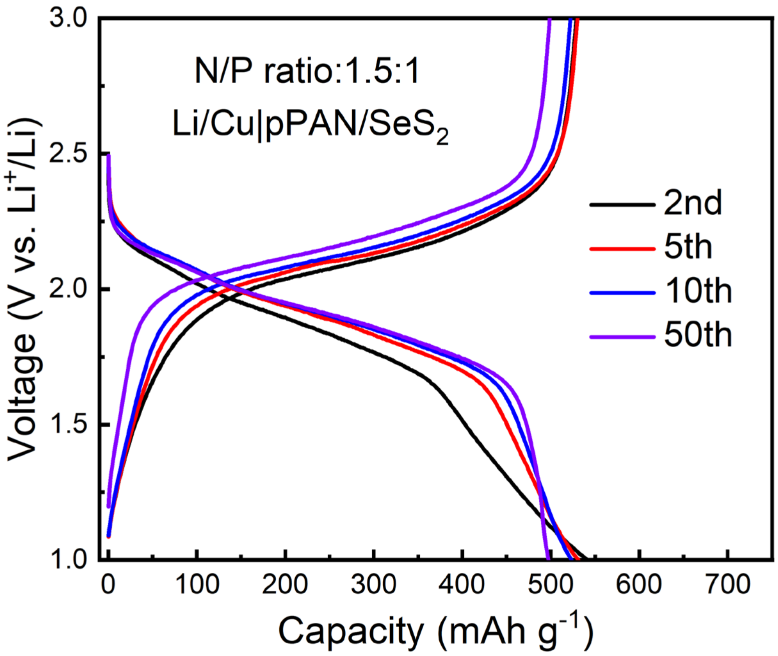

Figure S8. The discharge-charge curves of Li/Cu|pPAN/SeS_2 full-cell at 0.1 A g^{-1}.
